# High Output Piezo/Triboelectric Hybrid Generator

**DOI:** 10.1038/srep09309

**Published:** 2015-03-20

**Authors:** Woo-Suk Jung, Min-Gyu Kang, Hi Gyu Moon, Seung-Hyub Baek, Seok-Jin Yoon, Zhong-Lin Wang, Sang-Woo Kim, Chong-Yun Kang

**Affiliations:** 1 Electronic Materials Research Center, Korea Institute of Science and Technology (KIST), Seoul 136-791, Korea; 2 Department of Nanomaterials Science and Technology, University of Science and Technology (UST), Daejeon, 305-333, Korea; 3 School of Material Science and Engineering, Georgia Institute of Technology, Atlanta, Georgia 30332-0245, United States; 4 School of Advanced Materials Science and Engineering, SKKU Advanced Institute of Nanotechnology (SAINT), Center for Human Interface Nanotechnology (HINT), Sungkyunkwan University, Suwon 440-746, Korea; 5 KU-KIST Graduate School of Converging Science and Technology, Korea University, Seoul 136-701, Korea

## Abstract

Recently, piezoelectric and triboelectric energy harvesting devices have been developed to convert mechanical energy into electrical energy. Especially, it is well known that triboelectric nanogenerators have a simple structure and a high output voltage. However, whereas nanostructures improve the output of triboelectric generators, its fabrication process is still complicated and unfavorable in term of the large scale and long-time durability of the device. Here, we demonstrate a hybrid generator which does not use nanostructure but generates much higher output power by a small mechanical force and integrates piezoelectric generator into triboelectric generator, derived from the simultaneous use of piezoelectric and triboelectric mechanisms in one press-and-release cycle. This hybrid generator combines high piezoelectric output current and triboelectric output voltage, which produces peak output voltage of ~370 V, current density of ~12 μA·cm^−2^, and average power density of ~4.44 mW·cm^−2^. The output power successfully lit up 600 LED bulbs by the application of a 0.2 N mechanical force and it charged a 10 μF capacitor to 10 V in 25 s. Beyond energy harvesting, this work will provide new opportunities for developing a small, built-in power source in self-powered electronics such as mobile electronics.

Energy harvesting devices have been intensively investigated not only to cope with the global energy crises, but also to realize the self-powered electronics such as implanted medical devices and mobile electronics^1^,^2^,^3^,^4^,^5^. Mechanical energy is one of the largest sources of the wasted energy in the modern civilization. Therefore, a variety of approaches have been demonstrated to convert the mechanical energy into the electric energy using different mechanisms: for example, electrowetting^6^, electromagnetics^7^,^8^, magnetostriction^9^, piezoelectricity^10^,^11^,^12^,^13^,^14^, and triboelectricity^15^,^16^,^17^,^18^,^19^,^20^.However, there are several issues to be overcome for the application in the self-powered electronics. First, the output power of the state-of-art energy harvesters is still quite low, limiting the range of their applications. Second, the energy harvester has to be strong enough to endure long-time exposure to the vibrational motions. Third, the fabrication process has to be favorable in terms of the massive production. Finally, for harvesting energy from portable devices, it should be able to operate under small mechanical force, such as body movements. To date, it seems that any particular types of energy harvesters alone are not able to satisfy these requirements, yet.

Here, in order to address the above issues, we demonstrate a prototypical, piezoelectric/triboelectric hybrid generator that can produce high output power due to the cooperative operation of piezoelectric and triboelectric mechanisms in a single press-and-release cycle. We use organic materials to build the hybrid generator: organic ferroelectric polyvinylidene difluoride (PVDF) for piezoelectric generator and polytetrafluoroethylene (PTFE) film. To overcome a limitation of PVDF generator such as a low output power^21^,^22^,^23^,^24^,^25^, we have designed a novel piezoelectric generator with a high output current and integrated it into triboelectric generator which produces high output voltage. Moreover, our triboelectric generator does not have any nanostructured surface modification. This makes our hybrid generator more reliable in terms of device lifetime, and more industry-friendly in terms of fabrication process. Using two full-wave bridge rectifiers, we obtain peak output voltage, current density, and power density of ~370 V, 12 μA·cm^−2^, and 4.44 mW·cm^−2^, respectively. The electrical energy output of the hybrid generator successfully lit up 600 light-emitting diode(LED) bulbs by a force of fingers (~0.2 N).

## Results

**Figure 1:**
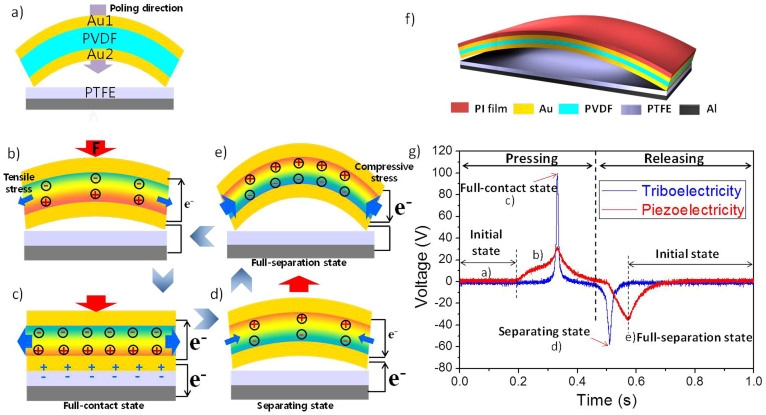
Working mechanism of the hybrid generator in a press-and-release cycle. (a) Initial state without a mechanical force. (b) Piezoelectric potential when the external force starts to be applied. (c) Piezoelectric and triboelectric charge distribution at full-contact state. (d) Negative piezoelectric and triboelectric generation at separating state. (e) Maximized negative piezoelectric potential at Full-separation state. (g) Schematic view of the arch-shaped piezo/triboelectric hybrid generator. (h) A graph of the piezo/triboelectric output voltages of the hybrid generator that are simultaneously measured in a single press-and-release cycle.

Figure 1(f) shows the 3-D schematic view of the arch-shaped piezo/triboelectric hybrid generator. The hybrid generator consists of vertically-stacked two layers: anarch-shaped piezoelectric generator with a Au/PVDF/Au stucture on the top, and a touch-and-releasetype triboelectric generator with PTFE/Al structure on the bottom. It has a common electrode shared as a bottom electrode of piezoelectric generator and as a driving electrode of triboelectric generator. The hybrid generator operates by the vertical force.

The top layer has a pre-strained piezoelectric PVDF generator with an arch shape supported by a polyimide (PI) substrate (Supplementary Fig. S1). Such an arch-shape of piezoelectric generator enhances the level of the effective strain applied in the PVDF layer as well as drives the shape of the generator to the original state upon the release of the applied force. The arch-shaped piezoelectric generator produces an electricity by d_31_ mode. The bottom layer consists only of a PTFE film coated with Al electrodes. It is noted that we have not fabricated any nanostructures on the surface of PTFE. To simultaneously apply the piezoelectric and triboelectric mechanisms, we use the bottom electrode of the piezoelectric generator as a driving electrode for triboelectric generator. The details of the theoretical model and working principle of piezoelectric generators that use a PVDF and triboelectric generators have been presented^26^,^27^,^28^,^29^.

Figure 1(a)–(e) shows the schematic illustration of operating mechanism of our hybrid generator. At the initial state before the contact of the two layers, neither piezoelectric nor triboelectric potential is present, as shown in Figure 1(a). When an external force starts to be applied on the top layer, the PVDF has a tensile stress and generates a positive piezoelectric potential between the Au1 electrode and Au2 electrode by its deformation and electrons flow from Au2 to Au1, as shown in Figure 1(b). Once the top layer touches the bottom layer (full-contact state), a physical contact between Au2 electrode and PTFE results in a charge transfer. Since the PTFE tends to gain electrons than the Au, electrons are transferred and remain there as electrostatic charges as shown in Figure 1(c). At the moment of the full-contact the piezoelectric and triboelectric outputs reaches the maximum and then decreases.

When the applied force is released (separating state), the arc structure starts to bounce back as shown in Figure 1(d); in such case, since the Au2 receives electrons back to be neutral charges and the PVDF have a compressive force, the hybrid generator produces both negative triboelectric and piezoelectric output voltages, and electrons flows from the Al and Au1 to the Au2.When the two layers are fully separated, there are no triboelectric potential because it becomes neutral charges (Figure 1(e)). The PVDF, on the other hand, generates the maximum piezoelectric output because of the maximum strain at the moment of initial state, and piezoelectric charges are slowly decreased. Then a full cycle is achieved, and it will go back to the equilibrium state. Therefore, we simultaneously obtained both piezoelectric and triboelectric output signals within one press-and-release cycle.

The graph of the piezoelectric and triboelectric output voltages corresponding to each state explains the generation mechanism of the hybrid generator, as shown in Figure 1(g). The output voltage for the triboelectric generator is a lot sharper than that of piezoelectric generator, and there is a delay in the peaks in the second half, which is due to the triboelectric charges are compensated faster than piezoelectric charges as long is still residual strain in the film during mechanical releasing.

We also confirm that the role of the curved PI substrate is very important for the pre-strained and arch-shaped piezoelectric power generation. To verify the role of the PI substrate, we experimentally investigated the open-circuit output voltage of the PVDF film (5 × 2 cm^2^) with and without the PI substrate (Supplementary Fig. S2).Without the PI substrate, the peak open-circuit voltage and peak short-circuit current of the PVDF were only ~0.15 V and 0.7 μA, respectively, whereas the PVDF with the substrate reached the open-circuit voltage of ~80 V and the short-circuit current of ~7.5 μA. The power densities of the PVDF film with and without the substrate were 0.0105 and 60 μW·cm^−2^, respectively. This is because the PVDF with the substrate only has compressive or tensile stress in whole volume during bending or releasing. However, without the PI substrate, extensive compressive and tensile stress occurs on the top and bottom of the PVDF at once during bending and releasing. For this reason the output power of the PVDF without the substrate was too small. This result demonstrated that the substrate significantly increased the output voltage of the PVDF.

When the generated power is measured separately, our piezo/triboelectric hybrid generator with a dimension of 7 × 3 cm^2^ can produce a piezoelectricity of ~80 V and 7.62 μA·cm^−2^and a triboelectricity of ~380 V and 4.3 μA·cm^−2^(Supplementary Fig. S3). However, when measured in the combined way, it can generate only ~120 V and 10 μA·cm^−2^, which was much lower than the expected output. This is attributed to the different internal resistances of piezoelectric and triboelectric generators. It is well known that the output voltage is dominant to the power source with a lower internal resistance in case of exsiting two power sources. This indicates that the piezoelectric generator has a lower internal resistance than the triboelectric one. In addition, in faster operating frequencies over ~7 Hz, the output power is degradated by the voltage cancellation due to the the phase difference between the piezoelectric and triboelectric output voltages (Supplementary Fig. S4).

**Figure 2:**
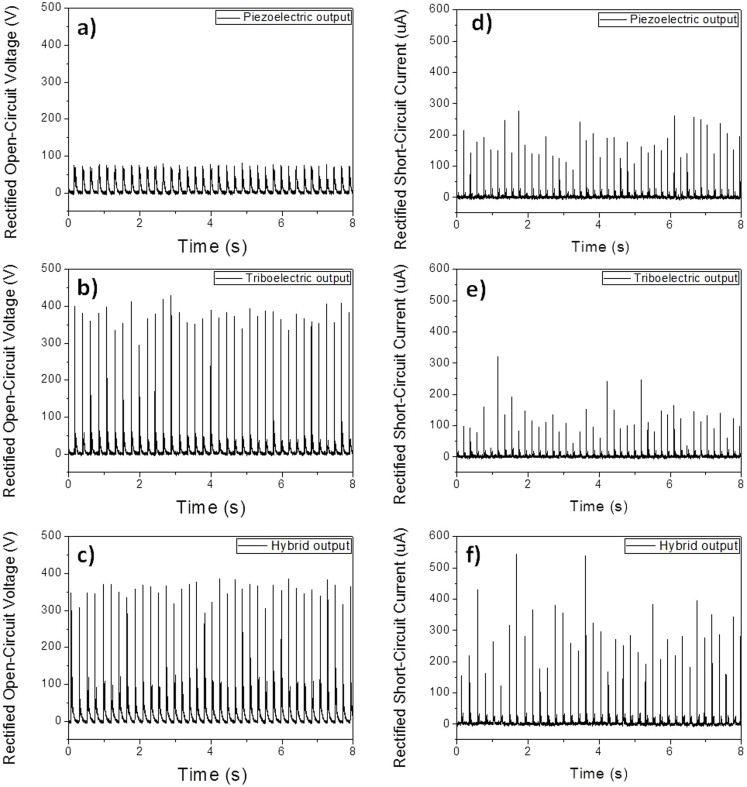
Open-circuit voltage and short-circuit current of the hybrid generator. (a,b) Simultaneously measured piezoelectric and triboelectric open-circuit output voltages. (c) Hybrid open-circuit output voltage. (d,e) Simultaneously measured piezoelectric and triboelectric short-circuit output currents. (f) Hybrid short-circuit output current.

To solve these problems, we utilized two full-wave bridge diodes that were independently connected to both the piezoelectric and triboelectric outputs (Supplementary Fig. S5). Figure 2 shows the rectified piezoelectric, triboelectric, and hybrid open-circuit voltages and short-circuit currents under a periodic mechanical force with the triggering frequency of 5 Hz. The rectified hybrid open-circuit output voltage was almost the same as the triboelectric output voltage because of the parallel connection of the piezoelectric and triboelectric output after rectification; the average peak output voltage was ~370 V, as shown in Figure 2(a)–(c). The hybrid output voltage properly combined the piezoelectric and the triboelectric output voltages, which is presented in detail in an enlarged view of the output profile (Supplementary Fig. S6). In the case of the rectified short-circuit output current, the piezoelectric short-circuit output current was much higher than the triboelectric short-circuit output current, and the hybrid output current is completely added to the piezoelectric and the triboelectric output, as shown in Figure 2(d)–(f). As a result, the hybrid open-circuit output voltage and current density were ~370 V and ~12 μA·cm^−2^, respectively, and the output power density was ~4.44 mW·cm^−2^, which is one order of magnitude higer than the previously reported r-shaped hybrid generator^30^. This result shows that the full-wave bridge diodes connected to the hybrid generator had effectively combined the piezoelectric and triboelectric output which resulted in a 2.5 times higher output power density than that before the rectification because of that full-wave bridge rectifiers does not allow voltage degradation by the differnect internal resistances of piezoelectric and triboelectric generators and eliminate the voltage cancellation effect. In addition, the rectification enables the hybrid generator to produce high output power regardless of the polarization direction of the PVDF film (Supplementary Fig. S7). We have investigated an external load matching for the hybrid generator. With an increase in the load resistance, the maximum otuput current decreased due to ohmic loss, whereas the maximum voltage has an opposite trend (Supplementary Fig. S8). Accordingly, the instantaneous output power caculated by W = I^2^_peak_ × R was ~0.62 mW·cm^−2^ at a load resistance of 30 KΩ.

## Discussion

**Figure 3:**
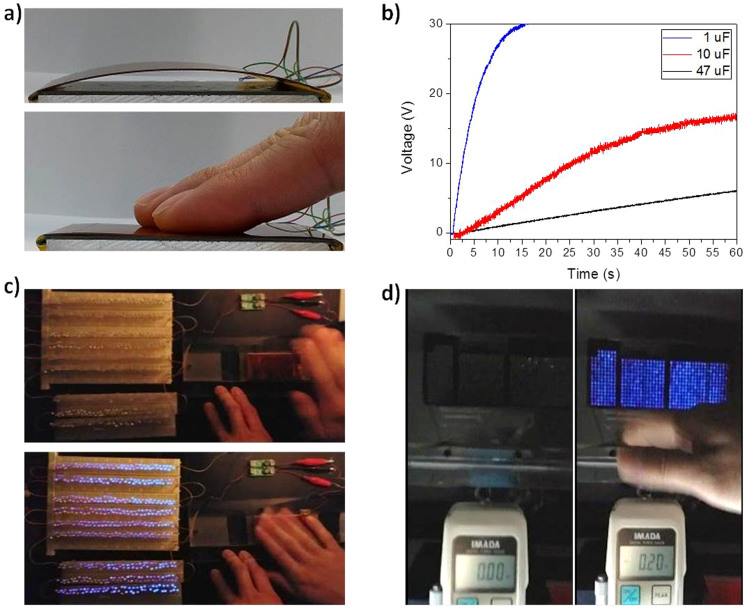
Output performances of the hybrid generator. (a) Photographs of the fabricated hybrid generator at the initial and full-contact states. (b) Charging voltage and time of the three capacitors with different capacitance values. (c) Snapshots of the 550 LED bulbs configured in series before and during the moment of being lit up. (d) Snapshots of the 600 LED bulbs configured in series and parallel before and during the moment of being lit up by a 0.2-N mechanical force.

Figure 3(a) shows a picture of the fabricated hybrid generator that the full-contact state can be easily achieved by a finger pressing due to arch-shaped structure. Some applications require a fast charging-and-discharging process or reach a higher voltage at a certain time, especially when a capacitor is chosen as a storage unit for a certain system. The charging of three capacitors with different capacitance values has been experimented, as shown in Figure 3(b). We found that the 1 μF capacitor was charged by the hybrid generator to 20 V in 5 s under periodic pressing and releasing (5 Hz).Also, the charging was 10 V in 25 s for the 10 μF capacitor and 5 V in 47 s for the 47 μF capacitor. Therefore, the proposed hybrid generator is powerful enough as an energy harvesting unit for power sources that employ mechanical force.

To demonstrate the electrical output performance of the hybrid generator, we utilized commercial LED bulb arrays to be lit up, which were connected in series and in parallel on LED panels, by calculating the hybrid output voltage and current. Figure 3(c) shows the snapshots of 550 LED bulbs (which were connected in series) before and during the moment of being successfully lit up in one full cycle (Supplementary Movie 1). Even a small ~0.2 N mechanical force, which was measured by a force meter, applied to the hybrid generator lit up 600 LED bulbs connected both in series and in parallel, as shown in Figure 3(d), (SupplementaryMovie2). In addition, the hybrid generator can light up to 880 LED bulbs with a maximum mechanical force (Supplementary Movie 3). Similar to the experiment of the load resistance, the output power varies as fucntion of the number of LED bulbs due tothe change of the resistance of LED bulbs (Supplementary Fig. S9). These results demonstrates that the proposed hybrid generator can be readily used in various applications such as a power source for wireless sensors or LED lighting for smart shoes, intelligent transport systems, and wireless switch for smart home.

Our hybrid generator has several advantages over the previously reported energy harvesters. First, it produces a high output power even at a mechanical force of as small as 0.2 N, which is capable of lighting 600 LEDs. Second, it can be fabricated at nominal cost and in large scale because it does not need nanostructures that still require a complicated process to fabricate. Third, as it consists of organic materials without any nanostructures, it will be more resistant to the mechanical failure. Fourth, it exhibits high current density owing to the use of both triboelectric and piezoelectric outputs. Compared with the piezoelectric nanogenerators, the proposed hybrid generator is superior in terms of not only the output voltage but also the output current. In addition, this hybrid generator is operated by a small mechanical force, compared with the previous piezoelectric energy harvesters.

The use of bridge diodes to prevent voltage cancellation leads to two advantages: one is the ability to obtain hybrid output voltage and current without a significant loss, and the other is that the polarization direction of the PVDF in the fabrication is not a concern. The hybrid output after rectification effectively combines the piezoelectric and the triboelectric output.

In conclusion, we demonstrate piezoelectric and triboelectric hybrid generator with a simple, prototypical structure using organic materials. It can generate an electricity of ~370 V of open-circuit voltage, ~12 μA·cm^−2^of output current density, and ~4.44 mW·cm^−2^of output power density by tapping it with fingers (~0.2 N). Such a large output power is enough to light 600 LED bulbs. Our hybrid approach will provide a framework to enhance the output power of the conventional power generators, and open a new avenue to realize self-powered energy systems such as mobile electronic devices.

## Methods

We purchased the PVDF with 0.1 mm of thickness from Fils co., LTD, the PI films with 0.25 mm of thickness from EDS system Inc., and PTFE film with 0.1 mm of thickness from EDS systems. The Au electrode with100 nm of thickness was deposited by on-axis mode with deposition rate of 0.2 nm/s, 5 × 10 − 6 Torr of the working pressure, 4.3 kV of the applying power, and 16.4 mA of current, whereas Al electrode with 200 nm of thickness was deposited on PTFE at room temperature for 5 min by DC sputter with 200 W of deposition power, 1 m Torr of working pressure, and 15 sccm of Ar flow rate. The total size of the hybrid generator was 7 × 3 cm^2^. The measurement of the output voltage and current was performed using the DPO 4014B Oscilloscope (Tecktronix) and SR570 low-noise current preamplifier (Stanford Research Systems), respectively. To measure open-circuit voltage and short-circuit current of the piezo/triboelectric hybrid generator, we have used the load resistance of 1 MΩ and 1Ω, respectively. Supplementary Fig. S10 shows the measurement configuration with those instruments. The pressing force measurement was conducted using a digital force gauge (IMADA Inc.).

## Author Contributions

W.S.J. conceived the idea, fabricated the device and wrote the main manuscript text. M.G.K. and H.G.M. conducted output measurements and prepared figures. S.H.B. analysed the data and contributed to manuscript preparation and S.J.Y., S.W.K. initiated and directed the research. C.Y.K. supervised the experiments and contributed to manuscript preparation. Z.L.W. provided advice for the research. All authors discussed the progress of research and reviewed the manuscript.

## Supplementary Material

Supplementary Information Supplementary Information

Supplementary Information Movie 1

Supplementary Information Movie 2

Supplementary Information Movie 3
