# Supplementary material for: High Output Piezo/Triboelectric Hybrid Generator
Source: Sci Rep. 2015 Mar 20;5:9309. doi: 10.1038/srep09309 (PMC4366849; doi:10.1038/srep09309)
Supplement: Supplementary Information [file srep09309-s1.docx]

**Supplementary information**

**High Output Piezo/Triboelectric Hybrid Generator**

Woo-Suk Jung^a^, Min-Gyu Kang^a^, Hi Gyu Moon^a^, Seung-Hyub Baek^a,b^, Seok-Jin Yoon^a^, Zhong-Lin Wang^c*^, Sang-Woo Kim^d*^, Chong-Yun Kang^a,e*^

^a^Electronic Materials Research Center, Korea Institute of Science and Technology (KIST), Seoul 136-791, Korea.

^b^Department of Nanomaterials Science and Technology, University of Science and Technology (UST),

Daejeon, 305-333, Republic of Korea

^c^School of Material Science and Engineering, Georgia Institute of Technology, Atlanta, Georgia 30332-0245, United States.

^d^School of Advanced Materials Science and Engineering, SKKU Advanced Institute of Nanotechnology (SAINT), Center for Human Interface Nanotechnology (HINT), Sungkyunkwan University, Suwon 440-746, Republic of Korea.

^e^KU-KIST Graduate School of Converging Science and Technology, Korea University, Seoul, 136-701, Korea.

^★^Corresponding author: [kimsw1@skku.edu](mailto:kimsw1@skku.edu), [zhong.wang@mse.gatech.edu](mailto:zhong.wang@mse.gatech.edu), [cykang@kist.re.kr](mailto:cykang@kist.re.kr)

Supplementary Movie 1. Lighting 550 LED bulbs with series connections during periodic pressing and releasing.

Supplementary Movie 2. Lighting 600 LED bulbs which are connected in series and parallel during periodic pressing and releasing.

Supplementary Movie 3. Lighting 880 LED bulbs using maximum mechanical force during periodic pressing and releasing.

Figure S1 | Configuration of the piezo/triboelectric hybrid generator. a) A top layer consists of PI substrate and PVDF film which Au electrodes are deposited by E-beam (pre-strained piezoelectric PVDF generator). b) A bottom layer has PTFE film with Al electrode. c) A photograph of the fabricated hybrid generator.

Figure S2 | Comparison of output voltage and current from the piezoelectric generator a,b) without a substrate and c,d) with the PI substrate.

Figure S3 | Output voltage and current of the hybrid generator before rectification. a) Measurement diagram for both piezoelectric and triboelectric outputs at the same time and b) for hybrid output. c) Piezoelectric and triboelectric open-circuit output voltages concurrently measured. d) Hybrid open-circuit voltage output. e) Piezoelectric and triboelectric short-circuit output current. f) Hybrid short-circuit output current.

**Figure S4** | **Comparison on piezoelectric and triboelectric outputs according to time interval between pressing and releasing.** a-c) No voltage cancelation. d-f) Voltage cancelation between positive piezoelectric output and negative triboelectric output.

Figure S5 | Circuit diagram of the piezo/triboelectric hybrid generator. a) Concurrent measurement method for piezo/triboelectric outputs. b) The hybrid output that the piezoelectric and triboelectric outputs are combined in parallel.

**Figure S6** | **Enlarged view of the rectified outputs from hybrid generator.** a) Piezoelectric and triboelectric open-circuit output voltages that are simultaneously measured. b) Hybrid open-circuit output voltage. c) Piezoelectric and triboelectric short-circuit output currents that are simultaneously measured. b) Hybrid short-circuit output current.

Figure S7 | a) Output voltage of the piezo/triboelectric hybrid generator with the opposite polling direction a) before rectification and b) after rectification. Inset: Enlarged view of the rectified output voltage.

Figure S8 | Dependence of a) output voltage and output current and b) output power on external load resistance.

Figure S9 | Measured voltage, current, and instantaneous output power as function of the number of LED bulbs


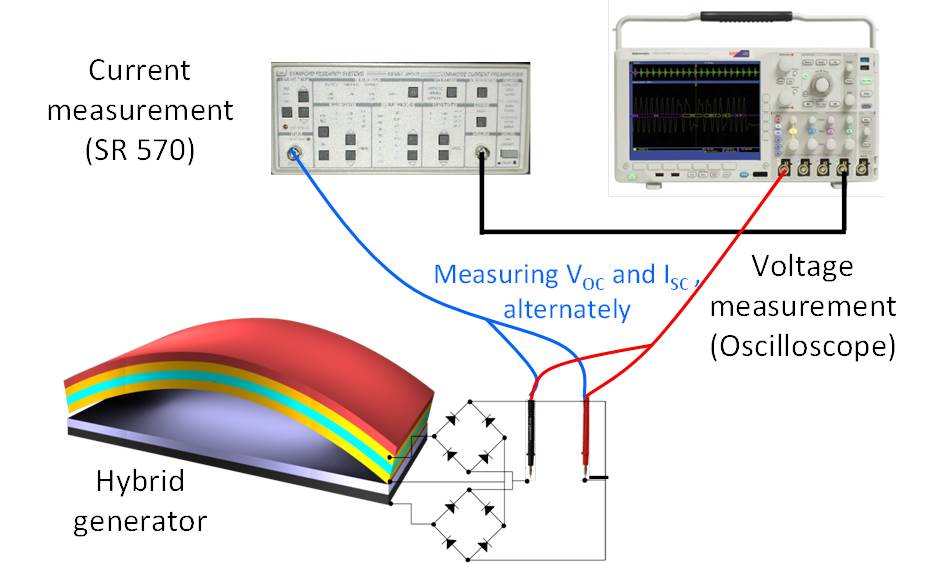


Figure S10 | Measurement configuration for piezo/triboelectric hybrid generator.
